# To eat or not to eat—an exploratory randomized controlled trial on fasting and plant-based diet in rheumatoid arthritis (NutriFast-Study)

**DOI:** 10.3389/fnut.2022.1030380

**Published:** 2022-11-02

**Authors:** Anika M. Hartmann, Melanie Dell'Oro, Michaela Spoo, Jan Moritz Fischer, Nico Steckhan, Michael Jeitler, Thomas Häupl, Farid I. Kandil, Andreas Michalsen, Daniela A. Koppold-Liebscher, Christian S. Kessler

**Affiliations:** ^1^Department of Dermatology, Venereology and Allergology, Charité—Universitätsmedizin Berlin, Corporate Member of Freie Universität Berlin and Humboldt-Universität zu Berlin, Berlin, Germany; ^2^Institute for Social Medicine, Epidemiology and Health Economics, Charité—Universitätsmedizin Berlin, Corporate Member of Freie Universität Berlin and Humboldt-Universität zu Berlin, Berlin, Germany; ^3^Department of Internal and Integrative Medicine, Immanuel Hospital Berlin, Berlin, Germany; ^4^Connected Healthcare, Hasso Plattner Institute, University of Potsdam, Potsdam, Germany; ^5^Department of Rheumatology and Clinical Immunology, Charité—Universitätsmedizin Berlin, Corporate Member of Freie Universität Berlin and Humboldt-Universität zu Berlin, Berlin, Germany; ^6^Department of Paediatric Oncology/Haematology, Otto-Heubner Centre for Paediatric and Adolescent Medicine (OHC), Charité—Universitätsmedizin Berlin, Corporate Member of Freie Universität Berlin and Humboldt-Universität zu Berlin, Berlin, Germany

**Keywords:** rheumatoid arthritis, fasting, caloric restriction, plant-based diet, inflammation

## Abstract

**Background:**

Fasting is beneficial in many diseases, including rheumatoid arthritis (RA), with lasting effects for up to 1 year. However, existing data dates back several decades before the introduction of modern therapeutic modalities.

**Objective:**

This exploratory RCT compares the effects of a 7-day fast followed by a plant-based diet (PBD) to the effects of the dietary recommendations of the German society for nutrition (Deutsche Gesellschaft für Ernährung, DGE) on RA disease activity, cardiovascular (CV) risk factors, and well-being.

**Methods:**

In this RCT we randomly assigned 53 RA patients to either a 7-day fast followed by an 11-week PBD or a 12-week standard DGE diet. The primary endpoint was the group change from baseline to 12 weeks on the Health Assessment Questionnaire Disability Index (HAQ-DI). Further outcomes included other disease activity scores, body composition, and quality of life.

**Results:**

Of 53 RA patients enrolled, 50 participants (25 per group) completed the trial and were included into the per-protocol analysis. The primary endpoint was not statistically significant. However, HAQ-DI improved rapidly in the fasting group by day 7 and remained stable over 12 weeks (Δ-0.29, *p* = 0.001), while the DGE group improved later at 6 and 12 weeks (Δ-0.23, *p* = 0.032). DAS28 ameliorated in both groups by week 12 (Δ-0.97, *p* < 0.001 and Δ-1.14, *p* < 0.001; respectively), with 9 patients in the fasting but only 3 in the DGE group achieving ACR50 or higher. CV risk factors including weight improved stronger in the fasting group than in the DGE group (Δ-3.9 kg, *p* < 0.001 and Δ-0.7 kg, *p* = 0.146).

**Conclusions:**

Compared with a guideline-based anti-inflammatory diet, fasting followed by a plant-based diet showed no benefit in terms of function and disability after 12 weeks. Both dietary approaches had a positive effect on RA disease activity and cardiovascular risk factors in patients with RA.

**Clinical trial registration:**

https://clinicaltrials.gov/ct2/show/NCT03856190, identifier: NCT03856190.

## Introduction

Rheumatoid arthritis (RA), one of the most common inflammatory diseases on a global scale, is associated with a high individual and socioeconomic burden (1, 2). Its etiology is considered multifactorial but remains largely unknown (3). Alongside a genetic predisposition, environmental factors seem to influence the onset and development of RA. This includes dysbiosis of the oral and intestinal microbiota, such as oral commensals like *Aggregatibacter actinomycetemcomitans*, which promotes citrullination of autoantigens, as well as colonic commensals like *Prevotella copri* (4). The theory of a gut-joint axis is supported by a growing body of literature on mechanisms of immune-mediated diseases (5–10).

In search for further non-pharmacological and cost-effective therapeutic approaches, nutrition has emerged as a relevant variable modulating the immune-system directly via pro- and anti-inflammatory food components as well as indirectly by influencing the gut microbiota toward an (anti-)inflammatory reaction (11, 12). Research on the effects of nutrition in RA suggests that certain dietary patterns, such as Mediterranean or pesco-vegetarian diets, may improve clinical symptoms of RA (13–16). Additionally, diet can positively influence common comorbidities of RA including risk factors determining morbidity and mortality, such as obesity, hypertension and dyslipidemia, as well as psychological conditions (17–20).

Recently, the French association for rheumatology has addressed the need for data-based dietary recommendations for rheumatologic patients (21). The society has presented a first set of dietary recommendations for French rheumatologic patients, essentially supporting weight loss for overweight patients, a Mediterranean-type diet and Omega-3 supplementation. According to this guideline, fasting and plant-based diets should not be proposed to patients. The authors conclude that vegetarian or vegan diets do not appear to have any relevant effects and question the sustainability of demonstrated fasting effects after resumption of the diet. This statement is based on three clinical fasting studies, the most recent dating back to 1991 (22), and six trials on plant-based diets dating back to 1979 (23).

Previous recent studies have indicated that caloric restriction may remarkably affect health, aging, and disease, including RA (24–26). Regarding RA, only the mentioned, almost historic clinical trials exist on the effect prolonged/periodic fasting (PF; <300 kcal/day) (22, 23). Despite the moderate study quality and age of the data, they show a significant positive impact of a 7 day fast on disease activity in RA for up to 1 year.

It has been hypothesized that a subsequent anti-inflammatory diet following fasting may sustain the beneficial effects beyond the days of the fast (27). A plant-based diet (PBD) seems to fit this demand best: it can reduce leukocyte counts (especially monocytes), can modulate inflammatory biomarkers such as CRP, possibly by altering the mTOR signaling pathway, and can positively impact gut microbiota (28–32).

While the recent extensive research on fasting and fasting mimicking diets is mostly experimental, most clinical trials on fasting or specific diets were performed before the widespread use of the new generation of disease-modifying antirheumatic drugs (DMARDs). Our objective was a) to generate a dietary therapeutic concept for patients with RA involving prolonged fasting followed by plant-based diet and b) to compare it in an explorative manner to an established guideline-based anti-inflammatory diet in patients with RA.

## Materials and methods

### Trial design

NutriFast was an open label, monocentric, randomized, controlled, parallel-group, explorative clinical trial. We registered the trial with ClinicalTrials.gov (NCT03856190) and published the study protocol in 2021 (33). The study was approved by the local ethics committee (EA 4/005/17) and conducted in accordance with the standards set out in the Declaration of Helsinki. All participants gave written informed consent prior to enrolment.

### Participants

We included patients of 18–79 years of age with RA diagnosed by a specialist. Eligibility criteria targeted patients on stable medication healthy enough to participate in an outpatient fasting program. Key exclusion criteria were arthritis other than RA, underweight, previous eating disorders, severe comorbidities, a strict PBD and/or fasting within the last 6 months or changes in therapy with DMARDs in the last 8 weeks before enrolment (33).

### Dietary intervention

The dietary interventions were delivered as an intensive group-based behavioral intervention in an outpatient setting and have been described elsewhere (33). In brief, the intervention consisted of a 7-day fast with a daily energy intake of 300–350 kcal/day through vegetable juices and vegetable broth. After fasting, participants were instructed to eat a subsequent plant-based diet for 11 weeks, integrating the concept of time-restricted eating (TRE) and anti-inflammatory spices. The control group followed the current dietary guidelines for RA of the German society for nutrition (Deutsche Gesellschaft für Ernährung, DGE) for 12 weeks. These recommendations are mainly based on a reduction in arachidonic acid intake, which is found in foods of animal origin.

### Outcomes

The primary outcome was the change in the Health Assessment Questionnaire Disability Index (HAQ-DI) after 3 months. Secondary outcomes regarding disease activity included Disease Activity Score 28 (DAS28), Simplified and Clinical Disease Activity Indices (SDAI, CDAI) and the proportion of participants fulfilling the EULAR response and American College of Rheumatology (ACR20, ACR50, ACR70) response. Furthermore, questionnaires were used to assess mood (Profile of Mood States, POMS), stress (Cohen's Perceived Stress Scale, CPSS-10), quality of life (WHO-5) and subjective intensity of the main complaint on a visual analog scale (VAS).

### Randomization and masking

We randomly assigned participants to the treatment arms in a 1:1 ratio. The randomization list was generated by an independent research team member using blockrand library (version 1.4) with a randomized variable block approach in R (version 3.5), consecutively numbered, sealed in opaque envelopes and concealed from the responsible study personnel. Participants were allocated after written informed consent and successful screening by the responsible study physician. Blinding of participants to the interventions was not possible due to obvious differences between the intervention arms. Similarly, blinding of outcome assessors was not feasible while ensuring medical and organizational support of patients in their specific dietary regimen.

### Sample size and power calculation

The sample size was calculated within the context of an explorative study. Estimating the yearly RA patient turnover of associated hospitals and clinics in Berlin, we intended to enroll 84 participants in this exploratory RCT. This number was based on a repeated ANOVA design with a hypothesis only for the interaction term, a non-sphericity correction of epsilon ε= 1, an assumed correlation among repeated measures of 0.5, and a dropout rate of 10%. The calculated sample size should provide us a solid statistical power (80%) with a significance level of 5% in detecting differences with effect sizes of f = 0.20 (d = 0.40) or higher, and thus all medium and large effects. In 12/2020, it became necessary to halt recruitment by 05/2021 due to mandatory research restrictions during the Covid-19 pandemic and expiring funding. Because the recruitment rate was slower than expected and recruitment ended early as mentioned, only 53 participants were enrolled.

### Statistical analysis

Analyses were based on the per-protocol treatment (PPT) population, excluding 3 early dropouts who did not start participation in either of the study interventions. Each analysis compared the outcomes between the two study arms before and after treatment. Potential differences in outcomes, were examined by χ2, analysis of variance, independent and dependent *t*-test as specified. We performed analyses using SPSS V.27 and RStudio Version 1.4.1106.

## Results

### Participant disposition and baseline characteristics

**Figure 1:**
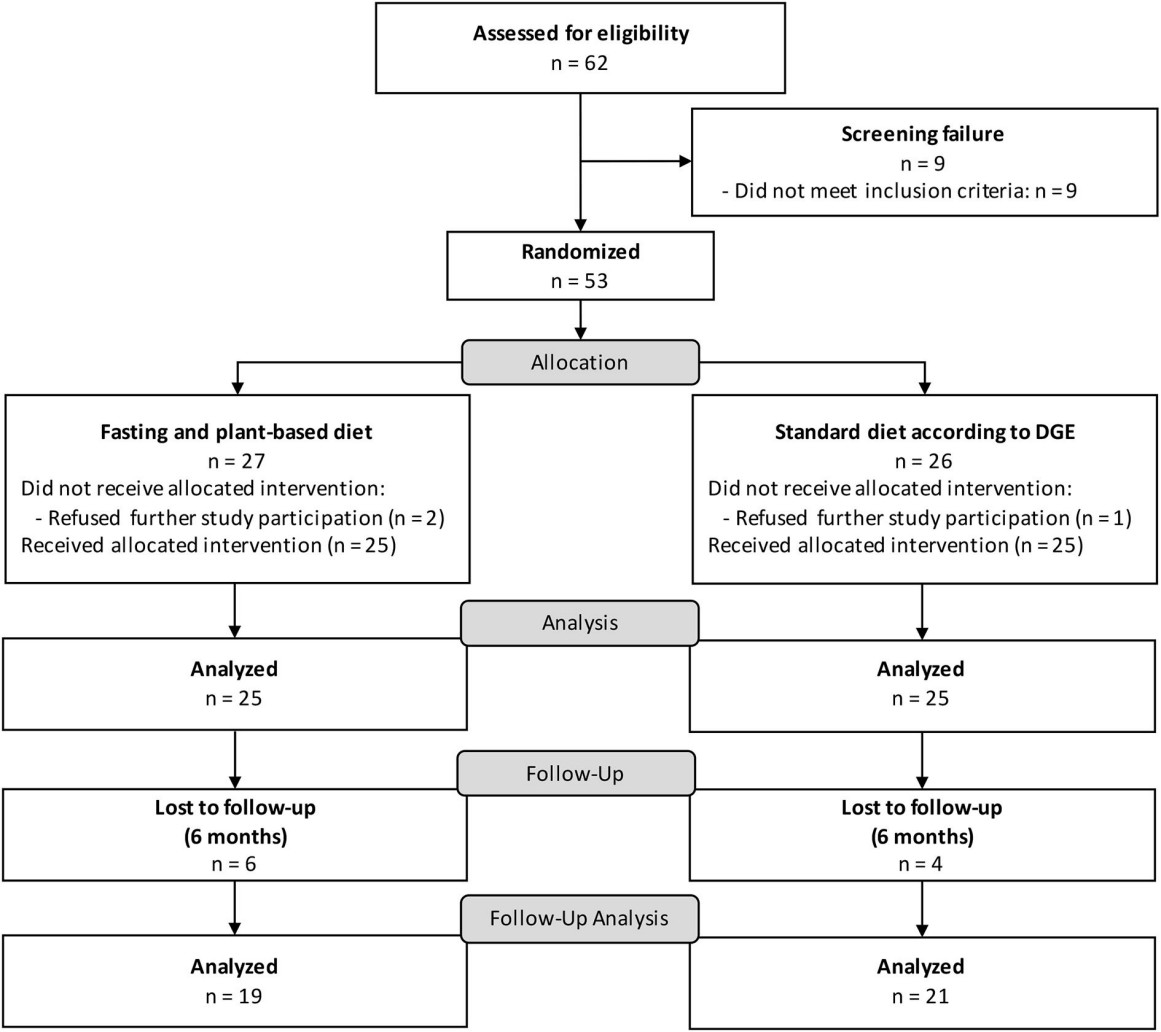
CONSORT flow chart NutriFast trial.

We enrolled 53 participants between 03/2019 and 11/2020 with follow-up at 6 months until 07/2021. Of 94 interested patients assessed per telephone, 62 patients were invited to a screening with a study physician, of which 53 fulfilled the eligibility criteria and were randomized to either fasting followed by a PBD or the standard diet according to the DGE recommendations (Figure 1). Fifty patients underwent the treatment per protocol, 3 early dropouts occurred before the start of the interventions. The follow-up at 6 months was completed by 40 participants. Demographics of the two groups were comparable at baseline and representative of a predominantly female RA population with a moderate disease activity (Table 1). Regarding baseline clinical characteristics, the fasting and PBD group demonstrated better functional ability in the HAQ-DI (Table 2).

**Table 1:** Demographic characteristics of participants at baseline.

	**Fasting and plant–based diet**	**Standard diet according to DGE**
	**(*****n** **=*** **25)**	**(*****n** **=*** **25)**
Age	52.8 ± 6.8	51.2 ± 11.5
Female sex	22 (88)	24 (96)
Body mass index, kg/m^2^	28.39 ± 5.11	27.28 ± 5.7
Current smokers	2 (8)	* 4 (16.7)
*Level of education*		
Upper secondary school	11 (44)	14 (58.3)
University degree or equivalent	14 (56)	10 (41.7)
*Occupational status*		*
Unemployed°	7 (28)	6 (25)
Working part time^+^	7 (28)	5 (20.8)
Working full time	11 (44)	13 (54.2)

Values reported are means ± SD or n (%) unless specified otherwise.

^*^of n = 24; ° including retirement, sick leave; ^+^ including students.

**Table 2:** Disease status and medication at baseline.

	**Fasting and plant–based diet**	**Standard diet according to DGE**
	**(*n =* 25)**	**(*n =* 25)**
Time since diagnosis, years^a^ Median (IQR)	3.30 (0.97;8.62)	3.33 (1.91;9.18)
RF and/or ACPA positive	18 (72)	21 (84)
RF positive	11 (44)	19 (76)
ACPA positive	13 (52)	16 (64)
HAQ–DI (0–3)^b^	0.69 ± 0.49	0.94 ± 0.54
DAS28–CRP	3.89 ± 1.26	4.03 ± 1.39
DAS28–ESR	4.19 ± 1.41	4.42 ± 1.58
SDAI	23.58 ± 14.83	26.16 ± 15.03
CDAI	20.75 ± 14.55	22.80 ± 14.22
In remission^c^	1 (4)	1 (4)
28–swollen joint count	5.22 ± 4.92	5.80 ± 5.54
28–tender joint count	8.72 ± 7.72	9.16 ± 7.73
Physician's global assessment of disease activity, 0–100 mm^d^	31.56 ± 23.35	36.32 ± 23.21
Patient's global assessment of disease activity, 0–100 mm^d^	36.53 ± 23.48	42.04 ± 26.54
C reactive protein, mg/L	2.84 ± 3.54	3.36 ± 4.01
ESR, mm/hour	15.08 ± 12.45	16.71 ± 11.77
Current RA treatment with csDMARDs	14 (56)	16 (64)
MTX	10 (40)	15 (60)
Sulfasalazine	3 (12)	2 (8)
Hydroxychloroquine	0 (0)	1 (4)
Leflunomide	3 (12)	2 (8)
Current RA treatment with bDMARDs	5 (20)	4 (16)
Adalimumab	0 (0)	1 (4)
Etanercept	3 (12)	1 (4)
Tocilizumab	2 (8)	0 (0)
Sarilumab	0 (0)	1 (4)
Rituximab	0 (0)	1 (4)
Current RA treatment with tsDMARDs	1 (4)	3 (12)
Baricitinib	0 (0)	3 (12)
Upadacitinib	1 (4)	0 (0)
Current treatment with glucocorticoids	6 (24)	9 (36)
Other medication		
NSAIDs	1 (4)	8 (32)
Azathioprin	1 (4)	0 (0)
Vitamin D derivates	12 (48)	7 (28)
Antihypertensives	4 (16)	1 (4)
PPI	2 (8)	4 (16)
Statins	0 (0)	2 (8)

Values reported are means ± SD or n (%) unless specified otherwise.

^a^Time since diagnosis of rheumatoid arthritis is presented as median and IQR.

^b^HAQ–DI scores range from 0 to 3, with higher scores indicating greater disability.

^c^Remission according to Boolean–based or index (SDAI)–based definition of remission developed by the American College of Rheumatology/European Alliance of Associations for Rheumatology.

^d^This evaluation is based on a VAS of 0–100, with higher scores indicating greater disease activity or pain.

ACPA, anti–citrullinated protein antibody; DGE, German society for nutrition; HAQ–DI, Health Assessment Questionnaire Disability Index; RF, rheumatoid factor; VAS, Visual Analog Scale; DMARD, disease–modifying antirheumatic drug.

### Primary outcome

**Figure 2:**
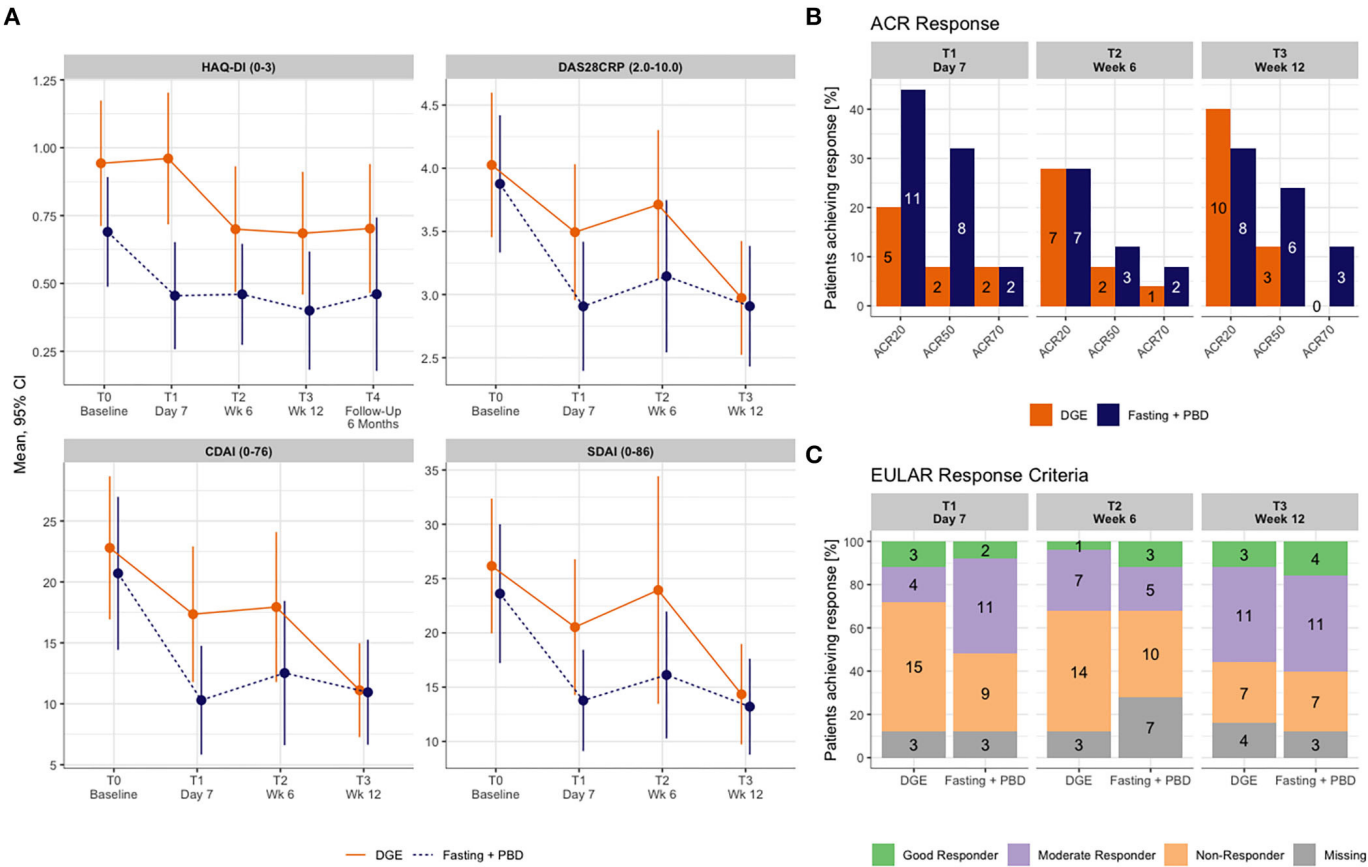
**(A)** HAQ-DI until 6 months and disease activity outcomes until week 12. **(B)** EULAR response until week 12. **(C)** ACR Response until week 12. ACR, American College of Rheumatology; CDAI, clinical disease activity score; CI, confidence interval; DAS28CRP, disease activity score 28 with CRP; DGE, Deutsche Gesellschaft für Ernährung (German society for nutrition); HAQ-DI, health assessment questionnaire disability index; PBD, plant-based diet; SDAI, simplified disease activity index.

Study outcomes are presented in Table 3 and in the Supplementary Appendix. The explorative primary endpoint, the between-group difference in the change from baseline to 12 weeks for the HAQ-DI, was not statistically significant (*p* = 0.663) (see Table 3). However, further analysis revealed a clinically relevant intra-group difference over time (Figure 2); the minimum clinically important difference (MCID) in RCTs currently being a change of 0.22–0.25 in the HAQ-DI (34, 35). The intervention group achieved a HAQ-DI reduction of-−0.24 ± 0.22 already at day 7 of the fast, which was sustained throughout the whole study period up until week 12 (Δ- 0.29 ± 0.38) and even to the 6-month follow-up (Δ-0.29 ± 0.37, Figure 2A). In the DGE group a similar functional enhancement was seen from study week 6 on (Δ-0.24 ± 0.49) up to 6 months (Δ-0.21 ± 0.44).

**Table 3:** Efficacy outcome change from baseline to day 7, week 6 and 12 for both treatment arms.

	**Fasting** + **PBD**			**DGE**			**Inter–group comparison day 7**	**Inter–group comparison week 6**	**Inter–group comparison week 12**
	**Change from baseline**			**Change from baseline**					
	Day 7	Week 6	Week 12	Day 7	Week 6	Week 12			
**Disease activity**							*p–value*	*p–value*	*p–value*
Δ HAQ–DI score	**−0.24** **±0.22**	**−0.23** **±0.31**	**−0.29** **±0.38**	−0.01 ± 0.37	**−0.24** **±0.49**	**−0.23** **±0.50**	**0.018**	0.936	0.663
Δ DAS28–CRP (2.0–10.0)	**−0.82** **±0.80**	**−0.65** **±0.96**	**−0.97** **±0.96**	**−0.53** **±1.05**	−0.37 ± 0.97	**−1.14** **±1.10**	0.298	0.362	0.568
Δ DAS28–ESR (2.0–10.0)	**−0.80** **±0.87**	**−0.52** **±0.98**	**−0.99** **±1.09**	−0.60 ± 1.39	−0.44 ± 1.04	**−1.13** **±1.24**	0.559	0.806	0.683
Δ SDAI (2.0–10.0)	**−8.18** **±8.89**	**−6.88** **±10.99**	**−10.09** **±9.68**	**−6.92** **±9.83**	−2.07 ± 19.91	**−12.53** **±12.74**	0.654	0.363	0.464
Δ CDAI (2.0–10.0)	**−8.77** **±8.39**	**−7.98** **±9.73**	**−9.41** **±9.20**	**−6.95** **±10.08**	−4.67 ± 10.93	**−12.44** **±13.01**	0.511	0.319	0.363
Δ 28–tender joint count	**−2.65** **±5.47**	**−2.89** **±5.22**	**−3.35** **±4.96**	**−2.44** **±5.49**	−1.52 ± 5.45	**−4.60** **±6.58**	0.894	0.413	0.463
Δ 28–swollen joint count	**−3.48** **±4.41**	**−2.39** **±4.16**	**−3.30** **±4.33**	**−2.76** **±5.36**	−1.28 ± 3.92	**−4.24** **±5.22**	0.617	0.378	0.505
Δ Patient's global assessment of disease activity, 0–100 mm	**−14.91** **±18.23**	**−13.78** **±21.42**	**−16.48** **±22.46**	−4.52 ± 18.99	−10.68 ± 22.48	**−19.08** **±28.79**	0.060	0.652	0.732
Δ Physician's global assessment of disease activity, 0–100 mm	**−11.52** **±23.18**	**−13.22** **±21.82**	**−11.09** **±18.25**	−0.36 ± 15.31	**−6.04** **±18.32**	**−12.83** **±21.68**	0.065	0.254	0.767
Δ CRP, mg/L	0.67 ± 3.08	1.04 ± 3.14	−0.66 ± 3.17	−0.06 ± 2.26	2.40 ± 12.52	4.55 ± 0.910	0.350	0.645	0.423
Δ ESR, mm/hour	1.73 ± 13.23	4.05 ± 10.78	−2.30 ± 7.87	1.80 ± 7.43	0.59 ± 11.23	2.36 ± 13.82	0.983	0.322	0.162
**Response criteria**									
*EULAR response criteria*									
Good responder	2	3	4	3	1	3	0.083	0.446	0.942
Moderate responder	11	5	11	4	7	11			
Non–responder	9	10	7	15	14	7			
*ACR response*									
ACR 70, *n* (%)	2/22 (9)	2/16 (13)	3/20 (15)	2/17 (12)	1/21 (5)	0/21 (0)	0.785	0.393	0.065
ACR 50, *n* (%)	8/22 (36)	3/16 (19)	6/21 (29)	2/17 (12)	2/21 (10)	3/21 (14)	0.081	0.416	0.259
ACR 20, *n* (%)	11/22 (50)	7/16 (43)	8/21 (38)	5/17 (29)	7/21 (32)	10/21 (48)	0.195	0.517	0.533

Values are based on a per–protocol analysis. Values are reported as the least–squares–mean change ± SD from baseline. Chi–Square–test was used to compare categorial variables, 2–sided t–test to compare continuous variables.

Since the primary endpoint was not met, all other p–values cannot be considered statistically significant and are presented for presentation only.

Significant values (p ≤ 0.05) of within–group mean change from baseline as well as significant p–values (p ≤ 0.05) of inter–group comparisons are bolded.

*Post-hoc* sensitivity analysis of the primary endpoint did not detect any correlations with antibody status, prior dietary habit, naturopathic treatment, and mode of dietary coaching (online vs. personal contact; see Supplementary File 1); however, data should be interpreted cautiously due to small subgroups.

### Secondary outcomes

#### Disease activity

Changes from baseline in disease activity followed a similar pattern as the primary outcome. DAS28CRP, DAS28ESR, SDAI and CDAI declined in the fasting and PBD group after 7 days and remained stable throughout the whole study period (Figure 2A). Disease activity in the DGE group declined by day 7, reverted slightly by week 6, and dropped significantly by week 12.

#### EULAR and ACR response rates

Fasting and PBD participants responded earlier to the dietary intervention (*n* = 13 of good and moderate EULAR response, *n* = 10 of ACR50 or higher response at day 7) compared with participants of the DGE group (*n* = 7 of good and moderate EULAR response, *n* = 4 of ACR50 or higher at day 7). By week 12, the fasting and PBD group still achieved a better response rate in the ACR response criteria (*n* = 9 and *n* = 3 with ACR50 or higher response, Figure 2B); however, by EULAR criteria, both intervention performed similarly (*n* = 15 and *n* = 14 with good and moderate EULAR response, respectively, Figure 2C).

#### Patient reported outcome measures

Lower scores in the profile of mood states indicated stabilization of emotions during the study period (Supplementary File 3, 4). Overall quality of life, measured by the WHO-Five Well-Being Index (WHO5), decreased by approximately 10 percentage points in the fasting and PBD group, with a significant difference from the DGE group at 6 months.

#### Laboratory assessment

Results of both groups indicated a trend toward decreasing CRP and ESR levels over 12 weeks (Table 3, Supplementary File 4). As previously described, liver enzymes, uric acid, and non-HDL cholesterol rose substantially during the fast and declined to normal levels after resuming a normocaloric diet, here by week 6 (Supplementary File 5). In the fasting and PBD group, eosinophiles and reticulocytes dropped during fasting (day 7) but returned to baseline values afterwards. Lymphocytes sank during the PBD while the leukocyte count remained stable. Vitamin B12 levels decreased in the fasting and PBD group within the reference range during the study period.

#### Cardiovascular risk and metabolic profile

**Figure 3:**
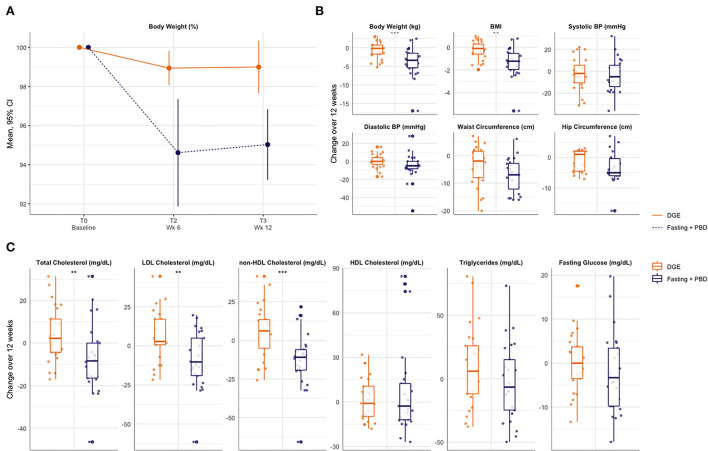
Cardiovascular risk profile through 12 weeks. **(A)** Relative change in body weight until week 12. **(B)** Change in clinical cardiovascular risk factors until week 12. **(C)** Change in laboratory cardiovaskular risk factors until week 12. BMI, body mass index; BP, blood pressure; DGE, Deutsche Gesellschaft für Ernährung (German society for nutrition); PBD, plant-based diet.

Participants in the fasting and PBD group achieved a clinically relevant weight loss (-4.5 kg) by week 6, which they maintained until the end of the study period (-3.9 kg), compared to the control group (−0.8 and −0.7 kg, respectively) (Figures 3A,B). Waist-to-hip-ratio (WHR) improved and both groups reached the reference range of normal weight with no significant between-group difference. Neither dietary protocol lowered systolic blood pressure nor heart rate within 12 weeks. Yet fasting seemed to favorably affect diastolic blood pressure (Figure 3B). Blood lipids and fasting glucose were also positively influenced by fasting and PBD by 3 months. Triglycerides, total, non-HDL- and LDL-Cholesterol were considerably reduced by week 6 through week 12. Fasting glucose considerably lowered within the standard range by day 7 of the fast and normalized through week 12 (Figure 3C, Supplementary File 5).

### Safety and medication

We observed one non-treatment emerged serious adverse event unlikely to be associated with the intervention (partial kidney resection) in the fasting and PBD group and no deaths in any of the groups (Supplementary File 6). Sixteen adverse events (AE) occurred in 10 participants (40%) undergoing fasting and PBD, and 9 AEs occurred in 6 participants (24%) consuming a DGE diet. No participants withdrew from the trial due to any AEs, which included diarrhea, paraesthesia, and fatigue. No major change in basic medication was made (Supplementary File 7).

## Discussion

In this study, we presented a dietary therapeutic approach for patients with RA involving prolonged fasting followed by a plant-based diet and evaluated its effects compared with an anti-inflammatory diet according to the DGE.

Regarding the primary endpoint, the change in HAQ-DI from baseline to 12 weeks, we found no significant inter-group difference of fasting followed by a plant-based diet to an anti-inflammatory guideline-based diet.

Having a look at within-group differences, fasting alleviated the subjective disease activity rapidly within 7 days. This gain was maintained through a PBD for up to 6 months and was not influenced by antibody status, delivery mode of the intervention or previous dietary patterns. The conventional guideline-based anti-inflammatory diet caused similar benefits by week 12, but at a slower rate. Regarding CV risk factors, fasting and PBD seem to reduce body weight and blood lipids in a sustainable way. Fasting and PBD resulted in a decrease of vitamin B12 levels within the reference range over 12 weeks.

We were able to observe improvement in RA disease activity by fasting which had been documented in previous trials (22, 23, 36, 37). At day 7, a clear difference to the anti-inflammatory DGE diet could be seen, which highlights the rapid onset of fasting effects. This characteristic suggests the suitability of fasting as a possible element of a short-term therapy i) for acute RA flares and ii) for patients preferring short-term rather than long-term changes in diet. Our data are consistent with other dietary interventions in RA, such as the ADIRA diet, which have led to similar effects in the mid-term above minimal important differences (MID) (13, 35).

Put into context of conventional therapies, we may have observed little effects on RA disease activity, possibly due to the comparatively strong impact of medication as well as an optimized lifestyle among our participants. This includes a previous diet including an already favorable omega-3 (Ω3) to omega-6 (Ω6) fatty acid ratio (38). Omega-3 fatty acids are known to reduce antibody-production in lupus-prone mice and inflammatory mediators (39, 40).

All-cause mortality among patients with RA is 54% higher than the general population, mostly due to CV disease. The underlying pathophysiological mechanisms of systemic inflammation in the CV system, such as elevated oxidative stress, endothelial dysfunction, and changes in lipid profiles, overlap with those of common CV risk factors and comorbidities of RA. For optimal CV disease management these risk factors need to be addressed. Here, patients undergoing fasting achieved clinically relevant weight loss and reduction in waist circumference which they maintained by a PBD including TRE. Owing to not measuring weight directly after fasting, we cannot differentiate between weight loss caused by prolonged fasting or PBD and TRE, both being known for their weight-lowering effectiveness. We observed ameliorated levels of atherogenic lipoproteins, as previously described by Grundler et al. (41). One could speculate whether further determinants such as the gut-derived metabolite trimethylamine N-oxide (TMAO) are favorably affected by long-term fasting and PBD. TMAO serum levels have been linked to CV disease and possible mechanisms such as impaired cholesterol transport, glucose tolerance, and insulin signaling (42). Intermittent fasting appears to reduce TMAO levels, either by diminishing the microbiota count or depriving TMAO precursors, namely animal-based protein (43, 44). These factors may also apply to long-term fasting and PBD.

A plant-based diet is known to lower vitamin B12 levels as it mainly occurs in animal products nowadays (45). Since vitamin B12 stores remain stable for up to several years, we did not substitute during the study and monitored serum levels after 12 weeks (46). As expected, these decreased significantly, but were still within the reference range. However, vitamin B12 would have to be substituted in the case of a permanent purely plant-based diet for more than 3 months.

Regarding inflammatory parameters, eosinophils sank while fasting as well as lymphocyte counts during the following PBD. The reduction in lymphocytes and white blood cells has also been documented in a randomized pilot-trial for a fasting-mimicking diet and ketogenic diet in patients with multiple sclerosis (47). This phenomenon falls in line with recent observations on T and B cells in murine models. Collins et al. demonstrated homing of memory T cells to the bone marrow in response to a 50% dietary restriction; hence plasma lymphocyte levels decreased, survival and protective function enhanced, and mTOR signaling declined (48). Choi et al. observed that a fasting mimicking diet increased the apoptosis rate of autoimmune T lymphocytes in a murine model for multiple sclerosis (26, 47). Nagai et al. reported on emptying B-cell pools in peyer's patches in short-term fasting either due to migration to the bone marrow (naive B-cells) or to apoptosis (germinal center and IgA+ B-cells). Upon refeeding, peyer's patches are replenished with naïve B-cells (49). Given that a vegan diet did not affect lymphocyte count in a study on a PBD in healthy participants, fasting rather than PBD might have caused this cell decline (28). Therefore, we speculate that dietary interventions, such as fasting, provide a supportive state for immune cells also in rheumatic diseases, although further investigation is required to test this hypothesis.

This study has several limitations. First, as most behavioral therapies, full blinding was not possible. For practical reasons, recruitment only took place in Berlin and surroundings, impairing generalization of our findings. Due to the Covid-19 pandemic and slower recruitment than expected we did not reach the calculated sample size. Both intervention groups consisted mainly of women, typical for RA but also a problem of dietary trials; our findings may therefore not apply to men with RA. The investigated population was healthy enough to complete an outpatient fasting program; hence our results might not be generalizable to more vulnerable individuals with RA, such as those having severe comorbidities and sarcopenia. Furthermore, we did not have any restrictions regarding disease activity, possibly resulting in a study population with too low or heterogeneous disease activity to achieve clinically relevant improvement. Despite randomization, the intervention groups differed significantly in their baseline HAQ-DI values, which is why we analyzed all outcomes regarding their performance over time. For ethical reasons and patient recruitment, we enrolled patients regardless of their pharmacologic therapies except that therapy had to have been stable for at least 8 weeks.

Lastly, both studied dietary forms may resemble each other to a certain extent, except from fasting, as both a plant-based diet and the DGE recommendations reduce the intake of AA. In previous studies other dietary concepts with reduced AA consumption alleviated RA symptoms, albeit not reaching statistical significance (13, 50).

In summary, our results show that both interventions, fasting followed by PBD and an anti-inflammatory DGE diet, improve functionality (HAQ-DI) and disease activity (DAS28/CDAI/SDAI) in RA and are not significantly different in the long term, but fasting acts faster. Relevant clinical improvements were seen within both dietary arms over the length of the study period, also in case of remote dietary counseling sessions. Hence, this trial supports the idea of dietary interventions as low-cost supportive elements to be included in an integrative therapeutic approach for patients with RA.

Further clinical studies are necessary to confirm our findings. A larger sample size and a more sensitive primary endpoint that could also be assessed after a longer follow-up period, e.g., one year, would be desirable. The study design could ideally include a third comparison arm that does not receive a therapeutic intervention. In addition, it would be of interest to incorporate patient treatment preference into the study design, as this could increase the attractiveness of study participation. It would also be interesting to investigate whether patients in an acute RA flare benefit from fasting.

## Conclusions

This exploratory study is a first approach to generate a dietary therapeutic concept for patients with RA involving prolonged fasting. There was no benefit of fasting followed by a plant-based diet regarding function and disability compared to a guideline-based anti-inflammatory diet after 12 weeks. However, fasting effects on disease activity set in more rapidly already by day 7. Both dietary concepts positively impacted RA disease activity and cardiovascular risk factors.

In clinical practice, some patients with RA are asking for ways to actively participate in treating their disease. Our data provide a starting point for confirmatory studies that need to validate such approaches with larger sample sizes. Further studies on the suitability of fasting as a possible element of a short-term therapy i) for acute RA flares and ii) for patients preferring short-term rather than long-term dietary interventions would be of scientific interest as well.

Because side effects of such dietary interventions are rare, the two nutritional approaches presented here can be offered to patients in clinical contexts. These can then be tested according to individual preferences and interest.

The results of this study can contribute to personalized medical approaches for patients with RA.

## Data availability statement

Data are available upon request. Requests on data sharing can be made by contacting the corresponding author. Data will be shared after review and approval by the trial scientific board, and terms of collaboration will be reached together with a signed data access agreement.

## Ethics statement

The studies involving human participants were reviewed and approved by Institutional Ethics Committee of Charité—Universitätsmedizin Berlin. The patients/participants provided their written informed consent to participate in this study.

## Author contributions

AMH, MD, TH, AM, DK-L, and CK designed the study. AMH, MD, MS, AM, DK-L, and CK developed the interventional concept. TH and AM procured funding. AMH, MD, MS, and JMF were responsible for patient recruitment and clinical data acquisition. AMH, NS, and FK analyzed the clinical data. AMH, MD, JMF, NS, MJ, FK, AM, DK-L, and CK interpreted the results. AMH wrote the initial draft of the manuscript and coordinated the editing process. MD, MS, JMF, NS, MJ, TH, FK, AM, DK-L, and CK contributed equally with edits, comments, and feedback. All authors read and approved the final manuscript.

## Funding

Supported by Corona Foundation (Corona Stiftung, Deutsches Stiftungszentrum Essen), grant number S199/10063/2016.

## Conflict of interest

The authors declare that the research was conducted in the absence of any commercial or financial relationships that could be construed as a potential conflict of interest.

## Publisher's note

All claims expressed in this article are solely those of the authors and do not necessarily represent those of their affiliated organizations, or those of the publisher, the editors and the reviewers. Any product that may be evaluated in this article, or claim that may be made by its manufacturer, is not guaranteed or endorsed by the publisher.

## Supplementary material

Supplementary File 1 *Post-hoc* sensitivity analysis of the primary endpoint regarding antibody status, prior dietary habit, naturopathic treatment, and mode of dietary coaching (online vs. personal contact).

Supplementary File 2 Patient reported outcome measures including WHO5, Cohen perceived stress scale and profile of mood states over 6 months.

Supplementary File 3 Patient reported outcome measures including WHO5, Cohen perceived stress scale and profile of mood states over 6 months.

Supplementary File 4 CRP levels, ESR levels, swollen and tender joint count over 12 weeks.

Supplementary File 5 Laboratory results over 12 weeks.

Supplementary File 6 Overview of adverse events.

Supplementary File 7 Medication during the study period.

## Abbreviations

ACR20/50/70: American College of Rheumatology response
AE: adverse event
CV: cardiovascular
CDAI: Clinical Disease Activity Index
CrP: c-reactive protein
CPSS-10: Cohen's Perceived Stress Scale
DGE: German society for nutrition (Deutsche Gesellschaft für Ernährung)
DAS28: Disease Activity Score 28
DMARD: disease-modifying antirheumatic drug
ESR: erythrocyte sedimentation rate
HAQ-DI: Health Assessment Questionnaire Disability Index
MCID: minimum clinically important difference
MID: minimal important difference
PBD: plant-based diet
RA: rheumatoid arthritis
POMS: Profile of Mood State
PPT: per-protocol treatment
RCT: randomized controlled trial
SDAI: Simplified Disease Activity Index
TMAO: trimethylamine N-oxide
TRE: time-restricted eating
VAS: visual analog scale
WHR: Waist-to-hip-ratio.
