# Supplementary material for: To eat or not to eat—an exploratory randomized controlled trial on fasting and plant-based diet in rheumatoid arthritis (NutriFast-Study)
Source: Front Nutr. 2022 Nov 2;9:1030380. doi: 10.3389/fnut.2022.1030380 (PMC9667053; doi:10.3389/fnut.2022.1030380)
Supplement: Supplementary File 1 — Post-hoc sensitivity analysis of the primary endpoint regarding antibody status, prior dietary habit, naturopathic treatment, and mode of dietary coaching (online vs. personal contact). [file Data_Sheet_1.zip › Supplementary Material/Appendix.pdf]

SUPPLEMENTARY INFORMATION

Supplementary file 1. Post-hoc sensitivity analysis of the primary endpoint regarding antibody status, prior dietary habit, naturopathic treatment, and mode of dietary coaching (online vs. personal contact).

Supplementary file 2. Patient reported outcome measures including WHO5, Cohen perceived stress scale and profile of mood states over 6 months.

Supplementary file 3. Patient reported outcome measures including WHO5, Cohen perceived stress scale and profile of mood states over 6 months.

Supplementary file 4. CRP levels, ESR levels, swollen and tender joint count over 12 weeks.

Supplementary file 5. Laboratory results over 12 weeks.

Supplementary file 6. Overview of adverse events.

Supplementary file 7. Medication during the study period.
