# Supplementary figures and images for: To eat or not to eat—an exploratory randomized controlled trial on fasting and plant-based diet in rheumatoid arthritis (NutriFast-Study)
Source: Front Nutr. 2022 Nov 2;9:1030380. doi: 10.3389/fnut.2022.1030380 (PMC9667053; doi:10.3389/fnut.2022.1030380)

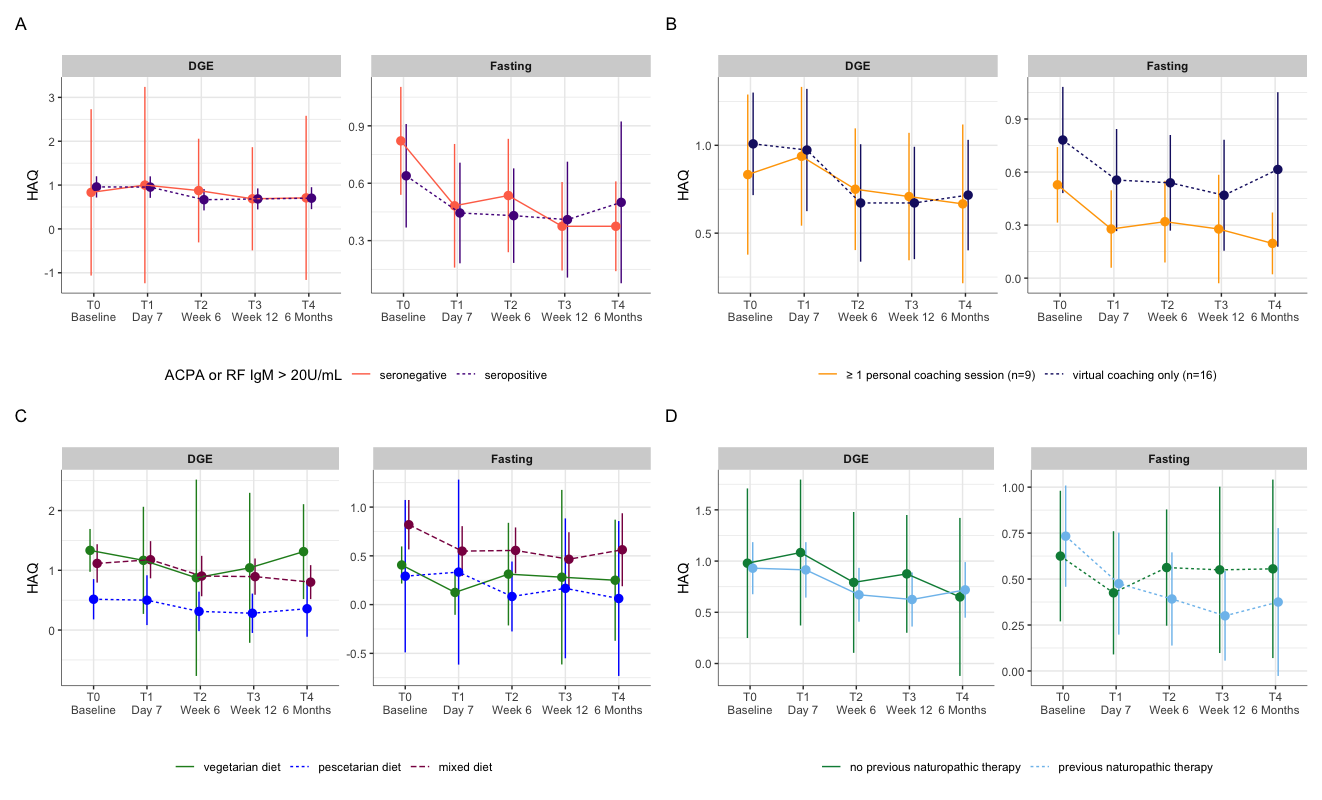

Supplement: Supplementary File 1 — Post-hoc sensitivity analysis of the primary endpoint regarding antibody status, prior dietary habit, naturopathic treatment, and mode of dietary coaching (online vs. personal contact). [file Data_Sheet_1.zip › Supplementary Material/SupplementaryFile_1.tiff]

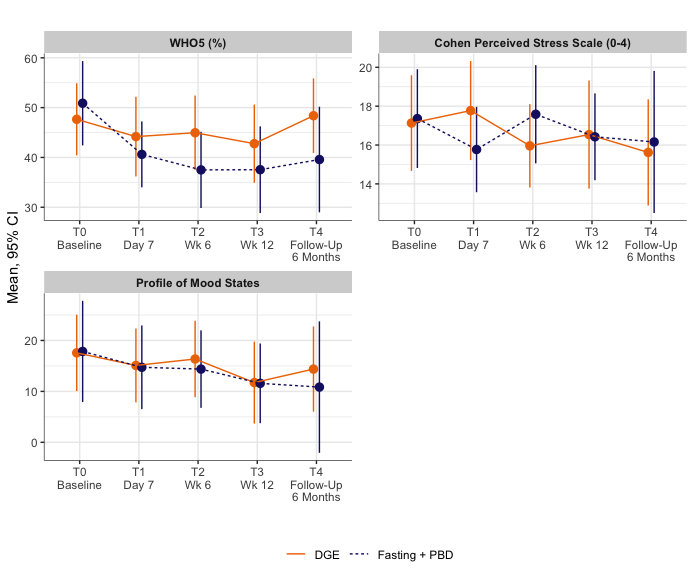

Supplement: Supplementary File 1 — Post-hoc sensitivity analysis of the primary endpoint regarding antibody status, prior dietary habit, naturopathic treatment, and mode of dietary coaching (online vs. personal contact). [file Data_Sheet_1.zip › Supplementary Material/SupplementaryFile_2.tiff]

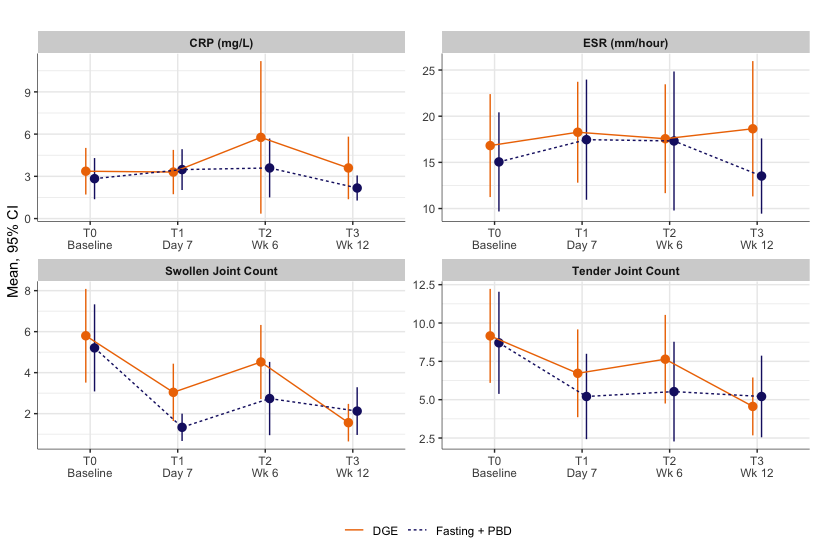

Supplement: Supplementary File 1 — Post-hoc sensitivity analysis of the primary endpoint regarding antibody status, prior dietary habit, naturopathic treatment, and mode of dietary coaching (online vs. personal contact). [file Data_Sheet_1.zip › Supplementary Material/SupplementaryFile_4.tiff]
